# Failed meniscal repair increases the risk for osteoarthritis and poor knee function at an average of 9 years follow-up

**DOI:** 10.1007/s00167-021-06442-w

**Published:** 2021-02-06

**Authors:** Erik Rönnblad, Björn Barenius, Anders Stålman, Karl Eriksson

**Affiliations:** 1 Stockholm Sports Trauma Research Center/Karolinska Institutet, Capio Artro Clinic, Valhallavägen 91, 114 86 Stockholm, Sweden; 2 Södersjukhuset/KISÖS, Stockholm, Sweden

**Keywords:** Meniscus repair, Meniscus injury, Osteoarthritis, Patient-reported outcome, ACL reconstruction

## Abstract

**Purpose:**

The purpose of this study was to determine the effect of meniscal repair on OA in the knee joint and patient-related outcomes.

**Methods:**

Three-hundred and sixteen meniscal repairs performed between 1999 and 2011 were analysed. Patient-related outcome measures were assessed through mailed questionnaires including KOOS, Lysholm score and Tegner activity level. Patients answering the questionnaires were encouraged to perform a radiographic evaluation with Rosenberg views, assessed according to Kellgren–Lawrence (KL) classification. The primary endpoint was to determine the effect of meniscal repair on the development of radiographic OA defined as a KL grade 2 or more.

**Results:**

Mean follow-up time was 9.3 years (SD 3.6), 162 (51%) patients answered the questionnaires, and 86 patients completed the X-ray. The odds ratio for OA with a failed meniscus repair was 5.1 (*p* = 0.007) adjusted for gender and age at time of follow-up. KOOS showed a clinically important difference in the sport and recreation subscale (*p* = 0.041).

**Conclusions:**

There was an increased risk for OA in the affected compartment with a failed meniscus fixation. This supports the fact that the meniscus is an important protector of the cartilage in the knee. The meniscus injury affects the long-term health-related quality of life according to KOOS and in light of this study we recommend repair of a torn meniscus whenever possible.

**Level of evidence:**

III.

## Introduction

Meniscal resection has been reported to increase the risk for osteoarthritis (OA) and reduced knee function [2, 12, 15, 32]. In patients with an anterior cruciate ligament (ACL) injury undergoing surgical reconstruction, meniscus injuries are reported in up to 40% [20] and concurrent resection of meniscal tissue is reported to have detrimental effects on postoperative knee function [28, 35, 39, 40]. In a recent publication, Cristiani et al. reported similar results in the Knee Injury and Osteoarthritis Outcome Score (KOOS) for isolated ACL reconstructions (ACLR) and ACLR in combination with meniscus resection or repair at 1- and 2-year follow-up [7]. However in a long-time follow-up at a mean of 14 years, Barenius et al. reported that in the ACL reconstructed knee, meniscus resection increases the risk for OA compared to meniscus repair [3]. The purpose of this study was to determine the effect of meniscal repair on OA and patient-related outcome for both isolated meniscal repair and repairs performed in conjunction to associated ligament reconstructions.

It was hypothesized that a successful meniscal repair will result in a lower risk for OA compared to a failed meniscal repair. It was also hypothesized that patients with successful meniscal repairs will have better subjective knee function that those with a failed meniscal repair.

## Materials and methods

The study was approved by the regional ethics committee (Karolinska Institutet, Sweden. ID number: 2014/689-31/3).

Patients who had a meniscal repair of a longitudinal, vertical tear, performed during 1999–2011 were identified retrospectively and medical charts were reviewed. Patient characteristics and surgical data including associated injuries and surgical procedures was collected. Patients were contacted through mail and asked to participate in the study. A written consent was requested and sent back together with the questionnaire in an attached envelope. Two reminders were sent out to reduce the number of loss to follow-up.

### Patient-related outcome

All patients were asked to complete a questionnaire including patient-related outcome measures. Subjective knee function was assessed using the Knee Injury and Osteoarthritis Outcome Score (KOOS) [34]. Patients were also asked to rate their knee according to Lysholm score and Tegner activity level.

### Radiographic assessment

All patients who accepted to undergo radiological examination had a weightbearing anterioposterior (AP) view taken with the knee joint in 30° of flexion (Rosenberg view) [36]. The radiographs were assessed according to the Kellgren–Lawrence (KL) classification [18] by the senior authors (KE and BB). In situations of uncertainty or when the senior authors disagreed on classification a radiologist was consulted. OA was classified as KL ≥ 2 (i.e. cartilage reduction ≤ 50% and/or significant osteophytes).

The primary endpoint was to determine the effect of meniscal repair on the development of OA. The second endpoint was to evaluate the effect of meniscal repair on subjective knee function.

Failure of meniscal repair was defined as symptoms requiring a subsequent partial or total meniscectomy in line with a previous study [33].

### Statistical analysis

Statistical analyses were conducted using IBM SPSS Statistics version 23 (SPSS Inc, Armonk, New York, USA). Statistical significance was considered at *p* < 0.05.

Categorical variables were tested using the Chi-square test, and continuous variables were tested using the independent *t* test.

Mann–Whitney *U* test was used for ordinal or non-parametric variables.

Logistic regression analysis was used to estimate the risk for OA between the successful meniscus repair group and the failed meniscus repair group. Odds ratios (OR) presented with 95% confidence intervals (CI) were used to estimate risk. If OR is > 1 the risk is higher than the reference group and vice versa. The deviation from 1 is considered significant at the 5% level if the CI does not include 1. After a univariate analysis, variables with a *p* value < 0.1 were included in the multivariate analysis.

To compare the KOOS subscale scores between the two meniscal repair groups, an analysis of covariance was used. Age at follow-up and gender were used as covariates. Age at follow-up was not significant after the univariate analysis and was therefore not included in the final model.

It was assumed that 20% of the patients with a successful meniscal repair should have osteoarthritis and 45% of the patients in the group with a failed repair. Based on a significance level of 5%, a power of 85 percent, and an effect size of 0.544, 72 patients should be included in each group.

## Results

A total of 318 patients were eligible for follow-up. Two patients had meniscal repairs performed in both knees and only the first surgical procedure was included in the analysis. The total failure rate of meniscal repairs in this cohort was 23.7 percent. Medial meniscal repairs had significantly more failures than lateral (*p* < 0.001). Meniscus repair with arrows had significantly more failures than repair with anchors (*p* = 0.011). A sub-analysis between patients with an isolated meniscus repair or a meniscus repair and an ACLR revealed significantly less failure in the meniscus repair and ACLR group (*p* = 0.041).

The mean follow-up time was 9.3 years (SD 3.6). The baseline characteristics are presented in Table 1.

**Table 1:** Demographic characteristics

	Total (*n* = 316)	Failed fixation		*p* value
		No (*n* = 241)	Yes (*n* = 75)	
Age at surgery				
Mean ± SD, yr	27 ± 9	27 ± 9	28 ± 9	n.s
FU time				
Mean ± SD, yr	9.3 ± 3.6	9 ± 3.7	10.4 ± 3.3	n.s
Sex				
Male	199 (63)	153 (76.9)	46 (23.1)	n.s
Female	117 (37)	88 (75.2)	29 (24.8)	
Meniscus				
Lateral	106 (33.5)	96 (90.6)	10 (9.4)	< 0.001*
Medial	197 (62.3)	136 (69)	61 (31)	
Both	13 (4.1)	9 (69.2)	4 (30.8)	
Repair method				
Anchor	147 (46.5)	123 (83.7)	24 (16.3)	0.011*
Arrow	163 (51.6)	113 (69.3)	50 (30.7)	
Both	6 (1.9)	5 (83.3)	1 (16.7)	
ACL				
No ACL injury	131 (41.5)	96 (73.3)	35 (26.7)	n.s
ACL injury, not simultaneously reconstructed	139 (44)	104 (74.8)	35 (25.2)	
Simultaneous ACL reconstruction	46 (14.6)	41 (89.1)	5 (10.9)	

Data are reported as number (percentage) unless otherwise indicated

*ACL* anterior cruciate ligament, *n.s.* non-significant, *SD* standard deviation, *yr* years

*Statistically significant (*p* < 0.05)

### Patient-reported outcome

**Fig. 1:**
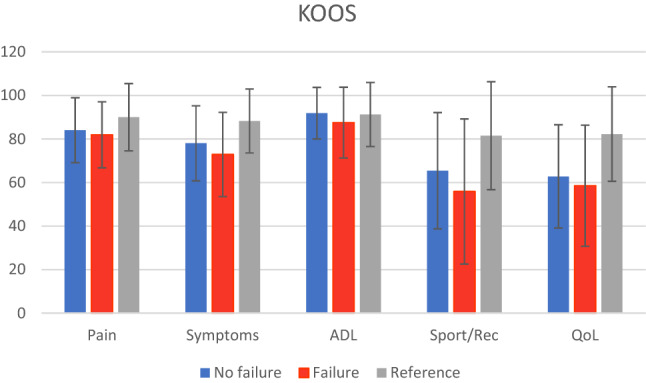
Knee Injury and Osteoarthritis Outcome Score (KOOS) subscales for failed and not failed meniscal repair. Mean KOOS values are shown for no failure (blue bar), failure (red bar), and a reference population of 18–54-year-old men and women (grey bar) from Paradowski et al. [29]

A total of 162 (51%) patients answered the questionnaires. There was a significant difference between the meniscus status groups in the KOOS Symptoms (*p* = 0.009), ADL (*p* = 0.020), and Sport/Rec (*p* = 0.041) subscales, in favor of successful meniscal repair. There were also significantly better results in Lysholm for the successful repair group (*p* = 0.036). Results are detailed in Table 2. For the KOOS values the greatest difference was found in the Sports/Rec subscale with eleven points, presented graphically in Fig. 1.

**Table 2:** Distribution of demographics and KOOS outcome score and Lysholm score depending on meniscus repair status

	Failed fixation		*p* value
	No 126 (77.8)	Yes 36 (22.2)	
Age at FU			
Mean ± SD, years	38 ± 12	41 ± 10	n.s
FU time			
Mean ± SD, years	8.9 ± 3.7	9.1 ± 3.4	n.s
Sex			
Male	72 (79.1)	19 (20.9)	n.s
Female	54 (76.1)	17 (23.9)	
Meniscus			
Lateral	45 (93.8)	3 (6.2)	0.005*
Medial	76 (71.7)	30 (28.3)	
Both	5 (62.5)	3 (37.5)	
Repair method			
Anchor	55 (78.6)	15 (21.4)	n.s
Arrow	66 (76.7)	20 (23.3)	
Both	5 (83.3)	1 (16.7)	
ACL			
No ACL injury	47 (75.8)	15 (24.2)	n.s
ACL injury, not simultaneously reconstructed	57 (75)	19 (25)	
Simultaneous ACL reconstruction	22 (91.7)	2 (8.3)	
KOOS			
Mean ± SD			
Symptoms	78.2 ± 17.2	71.7 ± 20.5	0.009*
Pain	84.2 ± 14.9	82.2 ± 14.8	n.s
ADL	91.9 ± 11.9	85.6 ± 20.6	0.020*
Sport/Rec	65.7 ± 26.8	54.5 ± 33.2	0.041*
QoL	63 ± 23.8	57.5 ± 27.8	n.s
Lysholm	80.2 ± 16	73.3 ± 20.6	0.036*

Data are reported as number (percentage) unless otherwise indicated

*ACL* anterior cruciate ligament, *ADL* activities of daily living, *FU* follow-up, *KOOS* Knee Injury and Osteoarthritis Outcome Score, *QoL* quality of life, *n.s.* non-significant, *SD* standard deviation, *Sport/Rec* sport and recreation

*Statistically significant (*p* < 0.05)

### Osteoarthritis

Eighty-six patients completed the radiographic investigation. The distribution of patients with OA and meniscal repair status is presented in Table 3. All patients but three who underwent radiological examination had also answered the questionnaire. In total, 26.7% had developed OA in the index operated compartment, i.e. the same compartment as the meniscal repair. Older age at the time of follow-up increased the risk for OA with an OR of 3.818 (*p* = 0.024) and failure of meniscal repair increased the risk for OA with an OR of 5.1 (*p* = 0.007). Logistic regression analysis presented in Table 4.

**Table 3:** Distribution of OA depending on meniscal repair status

	Failed fixation		*p* value
	No 68 (79.1)	Yes 18 (20.9)	
Age at FU			
Mean ± SD, yr	37.2 ± 11.8	40.5 ± 10.3	n.s
FU time			
Mean ± SD, yr	8.9 ± 3.7	9.1 ± 3.4	n.s
Sex			
Male	35 (81.4)	8 (18.6)	n.s
Female	33 (76.7)	10 (23.3)	
Meniscus			
Lateral	26 (92.9)	2 (7.1)	0.048*
Medial	40 (74.1)	14 (25.9)	
Both	2 (50)	2 (50)	
Repair method			
Anchor	34 (81)	8 (19)	n.s
Arrow	32 (78)	9 (22)	
Both	2 (66.7)	1 (33.3)	
ACL			
No ACL injury	22 (75.9)	7 (24.1)	n.s
ACL injury, not simultaneously reconstructed	31 (73.8)	11 (26.2)	
Simultaneous ACL reconstruction	15 (100)	0 (0)	
OA			0.007*
Yes	13 (56.5)	10 (43.5)	
No	55 (87.3)	8 (12.7)	

Data are reported as number (percentage) unless otherwise indicated. OA classified as KL ≥ 2 in index operated compartment. Adjusted for age and gender

*ACL* anterior cruciate ligament, *FU* follow-up, *n.s.* non-significant, *SD* standard deviation, *OA* osteoarthritis, *yr* years

*Statistically significant (*p* < 0.05)

**Table 4:** Logistic regression analysis of OA depending on meniscal repair status

	*B*	S.E	Sig	OR	CI
Gender	− 0.472	0.546	n.s	0.624	0.214–1.817
Age at FU > 34 yr	1.34	0.592	0.024*	3.818	1.197–12.181
Failed fixation	1.633	0.605	0.007*	5.118	1.563–16.762

Included in the analysis was gender, age and variables with a *p* < 0.1 from the univariate analysis

*FU* follow-up, *n.s*. non-significant, *yr* years

*Statistically significant (*p* < 0.05)

### Non-response analysis

A comparison between patients answering and patients not answering the questionnaire and patients undergoing and not undergoing radiologic examination is presented in Table 5.

**Table 5:** Patient characteristics for answerers and non-answerers of the questionnaire and X-rayed and not X-rayed patients

	Answered questionnaire		*p* value	X-ray		*p* value
	Yes (*n* = 162)	No (*n* = 154)		Yes (*n* = 86)	No (*n* = 230)	
Age at FU						
Mean ± SD, yr	38.8 ± 11.6	42.2 ± 11.5	n.s	38.7 ± 11.5	48.8 ± 15	n.s
FU time						
Mean ± SD, yr	9.3 ± 3.7	9.2 ± 3.4	n.s	9.2 ± 3.7	10.5 ± 2.5	n.s
Sex						
Male	91 (45.7)	108 (54.3)	0.010*	43 (21.6)	156 (78.4)	0.003*
Female	71 (60.7)	46 (39.3)		43 (36.8)	74 (63.2)	
Meniscus						
Lateral	48 (45.3)	58 (54.7)	n.s	28 (26.4)	78 (73.6)	n.s
Medial	106 (53.8)	91 (46.2)		54 (27.4)	143 (72.6)	
Both	8 (61.5)	5 (38.5)		4 (30.8)	9 (69.2)	
Repair method						
Anchor	70 (47.6)	77 (52.4)	0.036*	42 (28.6)	105 (71.4)	n.s
Arrow	86 (52.8)	77 (47.2)		41 (25.2)	122 (74.8)	
Both	6 (100)	0 (0)		3 (50)	3 (50)	
ACL						
No ACL injury	62 (47.3)	69 (52.7)	n.s	29 (22.1)	102 (77.9)	n.s
ACL injury, not simultaneously reconstructed	76 (54.7)	63 (45.3)		42 (30.2)	97 (69.8)	
Simultaneous ACL reconstruction	24 (52.2)	22 (47.8)		15 (32.6)	31 (67.4)	
Failed fixation						
No	126 (52.3)	115 (47.7)	n.s	68 (28.2)	173 (71.8)	n.s
Yes	36 (48)	39 (52)		18 (24)	57 (76)	

Data are reported as number (percentage) unless otherwise indicated

*ACL* anterior cruciate ligament, *FU* follow-up, *n.s.* non-significant, *SD* standard deviation

*Statistically significant (*p* < 0 .05)

Females were overrepresented both among patients that answered the questionnaire (*p* = 0.010) and among patients assessed with radiographs (*p* = 0.003). Meniscal repair with arrows were significantly more represented among responders of the questionnaire (*p* = 0.036).

## Discussion

The most important result of this study is the fivefold increase in risk for OA with a failed meniscal repair. Failed meniscal repair was also associated with worse subjective outcome in the KOOS Symptoms, ADL and Sports/Rec subscales as well as Lysholm. This supports our hypotheses and gives evidence to the fact that the meniscus is important for the protection of the cartilage and the function of the knee joint.

### Failure of meniscus repair

The overall failure rate of 23.7% is in line with previous publications [33]. Worth mentioning is the shift in surgical technique from the previous meniscal arrows to the modern all-inside devices during the timespan of our study. Medial meniscus repairs have significantly more failures than lateral.

Failure of meniscus repair in conjunction with ACLR are generally reported to be lower than isolated repairs [33]. This is probably due to the beneficial effect of ACLR, theoretically both because of unavoidable restrictions postoperatively and the abundance of healing factors during the surgical procedure. In the first analysis, no such association was observed. When performing a sub-analysis between isolated repair or repair in conjunction to an ACLR, there was, however, significantly less failure in the meniscus repair and ACLR group.

### Patient-reported outcome

Patient-reported knee function has been reported to be influenced by the status of the meniscus. Lutz et al. [22] report superior results on all KOOS subscales but QoL for meniscal repair compared to meniscectomy. In association to an ACLR, meniscus repair has been reported to contribute to both better and worse outcome compared to resection in short-term follow-up. Melton et al. [24] reported worse results in the IKDC for patients who underwent a meniscectomy in conjunction to an ACLR. In a publication by Svantesson et al. [41] patients with a meniscus repair performed concomitantly to an ACLR demonstrated worse KOOS values at 1-year follow-up and Lysholm at 6 months follow-up. Similarly, LaPrade et al. [21] reported worse results after ACLR and meniscus repair in the KOOS Symptoms and QoL subscales at 2-year follow-up. With the uncertainty of potential failures of meniscus repair in the mentioned studies, Cristiani et al. [7] presented a similar study but in addition created subgroups depending on successful or failed meniscal repair. They found no difference in any of the KOOS subscales at 1- and 2-year follow-up for successful meniscus repair in conjunction to ACLR but poorer results with a failed meniscus repair. Phillips et al. [30] did on the contrary find worse results in terms of KOOS for patients who in association to an ACLR had a meniscus resection compared to meniscus repair. In a study with similar follow-up time as the present, Kimura et al. [19] reported excellent results in terms of Lysholm for patients who had undergone a meniscal repair. The numbers in their study was, however, small.

In terms of subjective outcome after ACLR and meniscus pathology, the Sports and recreation and Quality of Life subscales are reported to be of greatest importance [26].

The results of this study indicate that a meniscus injury affects the patients’ ability to be active in sports 9 years after their meniscus injury according to KOOS. The group with failed meniscus repair had an average of 55 and the group without failure 66 which is more than the reported minimal clinically important difference (MCID) of eight points [3]. Compared to a reference population for a similar age group without knee problems described by Paradowski et al. [29], the meniscus injury affects the long-term health-related quality of life in the whole group presented in Fig. 1. KOOS symptoms subscale had the strongest correlation to failed meniscus repair, but only seven points difference which gives a questionable clinical relevance.

### Osteoarthritis

The beneficial effect of the meniscus on cartilage protection has previously been described [9, 10, 17, 23, 27, 38–40]. Already in 1948, Fairbank reported an increased risk for OA with meniscus resection [12].

Barenius et al. [3] reported that a medial meniscus resection increases the risk for OA with an OR of 4.8, and a lateral meniscus resection with an OR of 4.2, both compared to resection. This is in line with Meunier et al. [25], who identified the status of the meniscus as the most important factor for OA after an ACL injury.

In this study, a significantly higher risk for OA with failed meniscus repair on the medial meniscus was found. This is in contrast to most previous studies indicating the lateral meniscus to be of greater importance for the development of OA [5, 6, 11]. The latter is supported by the report that removal of the medial meniscus increases the contact stress by 100%, whereas removal of the lateral meniscus increases contact stress by 200–300% [13]. The numbers in our study are small when looking at the sub-analysis of failed repair of the medial versus lateral meniscus. Higuchi et al. [16] did, however, also find the medial meniscus to be of greater importance for the protection against OA. During standing and running much of the loading goes through the medial compartment [14]. Obviously, this depends on the individual mechanical alignment. In the present cohort, no alignment measurements were made. Furthermore the medial meniscus has been reported to be of importance for the anterioposterior stability in the ACL reconstructed knee [8]. This could potentially be an explanation for the importance of the medial meniscus on the development of OA.

### Non-response analysis

Fifty-one percent answered the questionnaire. This is a relatively large loss to follow-up, but still comparable to the numbers in the Swedish National Knee Ligament Register (SNKLR) at 2-year follow-up [1]. Women were overrepresented responders to the questionnaire and completed the radiological examinations to a greater extent than men. This is in line with a previous non-response analysis performed on the SNKLR [31]. Patients who answered the questionnaire and completed the radiological examinations were also younger than non-responders, though not statistically significant. This is conflicting compared to results from the SNKLR. There was a significant difference in the repair group for those who answered the questionnaire. This is assumed to have no clinical implication since follow-up time between the groups did not differ.

One limitation of this study lays in its retrospective chart analysis. Furthermore, only vertical, longitudinal ruptures were included, but there were no strict criteria for what size or vascularization zone of the meniscus injury was to be repaired and thus included in the study. No allocation between different interventions was performed. The comparison is based on failed and successful repairs; however, there might be several factors for the failures that have not been analyzed. An ongoing degeneration could result in less successful repair, and also affect future OA in the knee joint. Additionally, no strict postoperative rehab protocol was used. In terms of restrictions and assessment for return to sports, standardized criteria were used, but physiotherapists could use their own rehab protocol, a protocol that we did not have access to in many of the cases.

Another limitation is that we only analyzed charts from our hospital. Even though we know that most patients are prone to contact the same clinic again if some adverse event would occur, we cannot be certain of this. During such a long follow-up time, it is for example unavoidable that some patients move and therefore seek consultation somewhere else. The number of failures could, therefore, potentially be higher. The individuals who answered the questionnaire and performed an X-ray have given information about contact at any other hospital or clinic, but those who did not answer we cannot be sure about.

The search in the chart database was based on meniscus repair. There is a possibility that some of the patients have had a surgical procedure, such as meniscus resection, cartilage injury etc., in the opposite knee without us finding that in our scrutiny.

Furthermore, we had limited information on BMI and smoking in the study. BMI has been reported to be of importance for the development of OA [37]. Smoking has been reported to increase the risk for failure after meniscal repair [4].

There was no information about knee alignment in the study. This could potentially influence both failure of a repaired meniscus and the development of OA.

The loss to follow-up is also a limitation. Even though it is desirable to have more patients included, 50% loss to follow-up is somewhat expected given the comparison of register studies.

Even though meniscal repair normally increases surgical time and costs in the short perspective, the long-term benefits for individuals and society seems unquestionable with increased functional outcome as well as reduction of subsequent osteoarthritis.

## Conclusion

There was an increased risk for OA in the affected compartment, with a failed meniscus fixation. This supports the fact that the meniscus is an important protector of the cartilage in the knee. The failed meniscus repair is affecting the patients’ ability to be active in sports 9 years after their meniscus injury according to KOOS. The meniscus injury is a serious injury to the knee and in light of this study we recommend repair of a torn meniscus whenever possible.
